# Microwave Antenna System for Muscle Rupture Imaging with a Lossy Gel to Reduce Multipath Interference

**DOI:** 10.3390/s22114121

**Published:** 2022-05-29

**Authors:** Laura Guerrero Orozco, Lars Peterson, Andreas Fhager

**Affiliations:** 1 Department of Electrical Engineering, Chalmers University of Technology, 41296 Gothenburg, Sweden; andreas.fhager@chalmers.se; 2 MedTech West, Sahlgrenska University Hospital, 41345 Gothenburg, Sweden; 3 Sahlgrenska Academy, University of Gothenburg, 40530 Gothenburg, Sweden; peterson.lars@telia.com

**Keywords:** microwave imaging, medical diagnosis, antenna system, muscle rupture, prototype, multipath signal, image reconstruction

## Abstract

Injuries to the hamstring muscles are an increasing problem in sports. Imaging plays a key role in diagnosing and managing athletes with muscle injuries, but there are several problems with conventional imaging modalities with respect to cost and availability. We hypothesized that microwave imaging could provide improved availability and lower costs and lead to improved and more accurate diagnostics. In this paper, a semicircular microwave imaging array with eight antennae was investigated. A key component in this system is the novel antenna design, which is based on a monopole antenna and a lossy gel. The purpose of the gel is to reduce the effects of multipath signals and improve the imaging quality. Several different gels have been manufactured and evaluated in imaging experiments. For comparison, corresponding simulations were performed. The results showed that the gels can effectively reduce the multipath signals and the imaging experiments resulted in significantly more stable and repeatable reconstructions when a lossy gel was used compared to when an almost non-lossy gel was used.

## 1. Introduction

Microwave imaging techniques are beginning to see an increase in clinical research for different applications. These techniques include breast cancer imaging [1,2], stroke detection [3,4,5] and others. In sports, muscle injuries are an increasing problem, especially injuries to the four hamstring muscles (the biceps femoris caput longum, biceps femoris caput breve, semimembranosus and semitendinosus muscles) on the back of the thigh that act as extensors in the hip joint and flexors in the knee joint. Hamstring injuries are the most common muscle injuries among athletes because they are non-contact injuries [6] and take place during exercises when the muscle develops tension while lengthening [7]. They have been reported in numerous different sports, such as sprinting, running, soccer and gymnastics [8,9]. The prevalence of hamstring tears in recreational sports and non-sporting situations are not well defined [10]; however, some cases have been reported among the general population [11,12]. These injuries represent a major cause of time lost in sports [13]. The rehabilitation process can be frustrating for the patient because the symptoms often persist for a long time (healing time can reach up to 12 months in serious cases) and re-injuries are common [14].

The biggest problem is that there are no scientific studies that objectively follow the muscle healing process over time, from injury through all phases of muscle healing from the inflammation phase to the regeneration phase and the remodeling-maturation phase. Consequently, there is no general consensus on the time that the patient needs to wait until it is safe to exercise again [15]. Some studies have reported that almost one out of three hamstring strains recur and that many happen within 2 weeks of returning to sports [16], a time frame that would likely decrease if it were better understood when it would be safe to return to physical activity.

Imaging plays a key role in the diagnosis and management of athletes with muscle injuries. Magnetic resonance imaging and ultrasonography are the imaging modalities of choice [17]. However, magnetic resonance imaging is a scarce resource that is expensive and has long measurement times that can be uncomfortable for some patients [18]. The portability and availability of ultrasound imaging systems make them an attractive imaging modality that can be used for functional tests. A disadvantage of these systems is that their effectiveness is dependent on the operator experience [12]. The operator should be skilled in the technique and have detailed knowledge of compartmental muscle anatomy, as well as experience in assessing normal and abnormal muscle tissue during the healing phases. For this reason, the microwave imaging system could be a breakthrough. This system can be portable and low cost, meaning that it could even be used on-site by the medical team where athletes are training, which would lead to a faster and safer diagnosis without the need for a skilled operator. Moreover, there is a future prospective with which microwave imaging could be used to follow the injury healing process to allow the athlete a safe return to sports and to prevent recurring injuries or complications.

Microwave imaging diagnostic techniques exploit the dielectric contrast between tissues. This particular work aimed to exploit this contrast for muscle rupture detection. A ruptured muscle causes a bleed inside the muscle and the detection of the rupture can be performed by assessing the dielectric contrast between the blood and the surrounding muscle tissue [19,20]. The tissue contrast is visualized by reconstructed images that represent the dielectric tissue properties using microwaves that are transmitted through the muscle.

There are two main microwave image reconstruction techniques: tomographic and radar-based imaging. In tomographic imaging systems, quantitative images of the following dielectric properties are generated: permittivity ε and conductivity σ. Image reconstruction requires the solution of an inverse scattering problem, usually with computationally demanding methods [21]. Many groups have generated simulation results using tomographic techniques [22,23]. Furthermore, several approaches have advanced to phantom and clinical investigations [24,25,26,27].

Radar-based microwave imaging does not generate quantitative images of dielectric properties, but instead identifies and localizes strong scatterers. These systems exploit a time of flight analysis of measured signals in either mono-, bi- or multistatic antenna system configurations. Many different confocal microwave imaging algorithms have been proposed to solve this imaging problem, such as the Delay and Sum (DAS) [28,29] and Delay, Multiply and Sum (DMAS) [30] algorithms, are commonly used. In these methods, received signals from the object are synthetically focused, creating an image of the strong scatterers. The confocal microwave imaging technique has been studied in simulations and in phantom and patient examinations. For example, the works of Fear and Stuchly [31] show examples of the monostatic technique. A group led by Craddock at the University of Bristol showed image reconstruction in clinical trials [32]. The technique has also been studied clinically by other groups [33].

A particular challenge with the radar-based algorithms is that the measured microwave signals include early-time and late-time content: the early-time content is dominated by reflections from the skin and the late-time content contains the desired object response. Before any imaging is attempted, the early-time content must be removed as it usually has a significantly larger amplitude than the late-time object response. Otherwise, the desired object response easily drowns in the much larger skin reflection. Different methods can be applied to reduce the early-time content. The most common is the use of a priori measurements of a tissue, such as phantom, lesion-free or healthy tissue [30], and the use of adaptive filtering algorithms [34,35]. The use of a priori data for patient measurement may not always be possible as it requires measurements to be taken before and after the injury and subtracting the latter from the former. In that case, a filtering algorithm may be more suitable. For experimental investigations on tissue phantoms, it is usually possible to perform before and after measurements. All of these algorithms are similar in that they attempt to estimate the early-time skin response so it can be removed from the signals before reconstructing the image. Consequently, they are sensitive to measurement variability and noise in the scattering data. Even a small variability creates errors or uncertainties in the estimation and removal of the early-time signal that could also corrupt the late-time object response to be preserved. One important step to help mitigate these problems is to reduce the variability in the measurement data and to keep the signals free from undesired scattering that originates from sources other than the skin and the inside of the body. Signal artifacts could occur as reflections from other parts of the antenna system and the surrounding environment, direct coupling and multipath signals between antennae, etc. As these signals also tend to be large in amplitude, even very small variations from measurement to measurement can introduce somewhat unpredictable inconsistencies in the estimation of the early-time signals and result in the poor removal of skin reflections. As a result, image reconstruction becomes prone to containing artifacts, which is shown and discussed in this article.

In the works by Meaney et al. [36], they found that multipath signals are the most significant factor in the corruption of the measured microwave signals in their imaging system. In this context, “multipath signals” is a uniting term that refers to all parts of the signals that do not originate from the signals propagating through the object under investigation. Instead, these are contributions from waves taking alternative routes (such as surface waves), reflections and scattering from the antennae themselves, supporting structures, cables or the surrounding environment. It also includes cross-channel leakage in the electronic system [37]. Multipath signals cannot be filtered out easily as they all originate from the same signal source as the desired signals, are at the same frequency and only appear as interference. The problem with multipath signals is most pronounced in near-field applications in which the signals are heavily attenuated by tissue [37].

By immersing the antennae and objects under investigation in lossy coupling baths, consisting of saline–glycerine mixtures, unwanted multipath signals can be attenuated [38,39,40]. At the same time, the coupling medium provides impedance matching between the antennae and the objects to maximize the energy coupled into the object. The lossy bath also has an advantageous effect on the operating bandwidth of the antennae, which is broadened due to increased resistive loading. This enables the use of simple and low-profile antennae, such as monopoles or dipoles, in a wide frequency band [41].

A somewhat different approach to maximizing the energy that is coupled into the object and, at the same time, minimize the power radiating in other directions is to design both off-body and on-body antennae with a high directivity. With the main radiation lobe directed toward the body, multipath effects from waves traveling outside and around the imaging target are minimized. Sometimes, this property is quantified by the front to back ratio and in this case, a high front to back ratio was desired. There are several examples of such antenna designs [2,3,42,43,44,45,46]. These antennae usually have many advantages, such as broader bandwidths, unidirectional radiating patterns with high gain and small sizes so there is space for a sufficient number of antennae within the imaging system. Unfortunately, these antennae also come with the disadvantage of increased complexity and, with that complexity, a finer meshing and more computationally demanding simulation models [47,48]. Typically, this means that this type of antenna is less suited for quantitative tomographic algorithms, for which accurate numerical models are needed, and more suited for radar-based algorithms.

Simpler monopole antennae seem to be effective in applications that aim for quantitative image reconstruction [48,49,50] and recently, we developed a fast reconstruction algorithm based on the 2D discrete dipole approximation [48]. This algorithm exploits the use of low-profile monopole antennae, which are modeled very efficiently using the analytical expressions of a line source. A lossy coupling bath ensures a reduction in unwanted multipath signals, for example, in the form of scattering from the wall of the imaging tank and the antenna elements themselves.

In this work, one of the goals was to investigate the design principles of an antenna system that is adapted for muscle rupture imaging and, at the same time, use a lossy matching medium to minimize multipath signals so that monopole antennae can be used. In this paper, we present a new antenna design that makes use of a semisolid gel consisting of a mix of saline water and agar to recreate the effects of antennae in a lossy bath [38,40] without having to immerse the entire leg, for example, in a tank filled with liquid. The idea of using a gel as a coupling medium is not new. Previously, regular ultrasound gel has been used for microwave simulations of bone density in the leg [51]. This gel, however, is almost lossless and, therefore, it does not dampen multipath signals. Furthermore, it is not solidified, making it a bit messier to handle. On the other hand, to the best of our knowledge, the idea to use a lossy and semisolid gel to eliminate multipath signals is novel. The goal was not to completely eliminate multipath signals but to attenuate them sufficiently to avoid corruption in the measured signals and image reconstruction. An additional feature of using a lossy gel is the shielding effect that they have on antennae against external disturbances. We used the DMAS radar-based imaging method to investigate the effects of reduced multipath signals on image reconstruction.

The overall goal of this work was to show the proof of principle of this novel antenna design. However, we did not attempt to optimize the antenna performance, nor the imaging accuracy for different shapes, sizes and positions of the leg and blood tissue phantoms. These questions will be addressed in future work.

This paper is organized as follows. Section 2 describes the proposed antennae and antenna system design, together with the measurement and simulation setup. Section 3 shows the simulated numerical results and the results from phantom experiments, as well as image reconstructions using the phantom measurements. Finally, the results are discussed in Section 4 and the conclusions are presented in Section 5.

## 2. Materials and Methods

In this section, we describe the simulation model and experimental setup that were used to investigate the antenna design, which was based on monopoles immersed in a semisolid lossy gel. The antennae and antenna system design are explained and we describe the investigations that were performed to determine their effects on the wave propagation and the reduction in multipath signals using different conductivity in the gel. The aim was to determine what is needed to achieve sufficient multipath signal reduction in order to facilitate accurate and consistent image reconstruction. We also investigated how the signal levels inside the leg were affected by the conductivity of the gel and the method that was used to investigate these effects is also described in this section. Lastly, the image reconstruction method is described, together with the experiments and simulations that were used to assess the imaging performance.

### 2.1. Dielectric Properties of Tissue, Phantom Materials and Simulation Models

**Figure 1:**
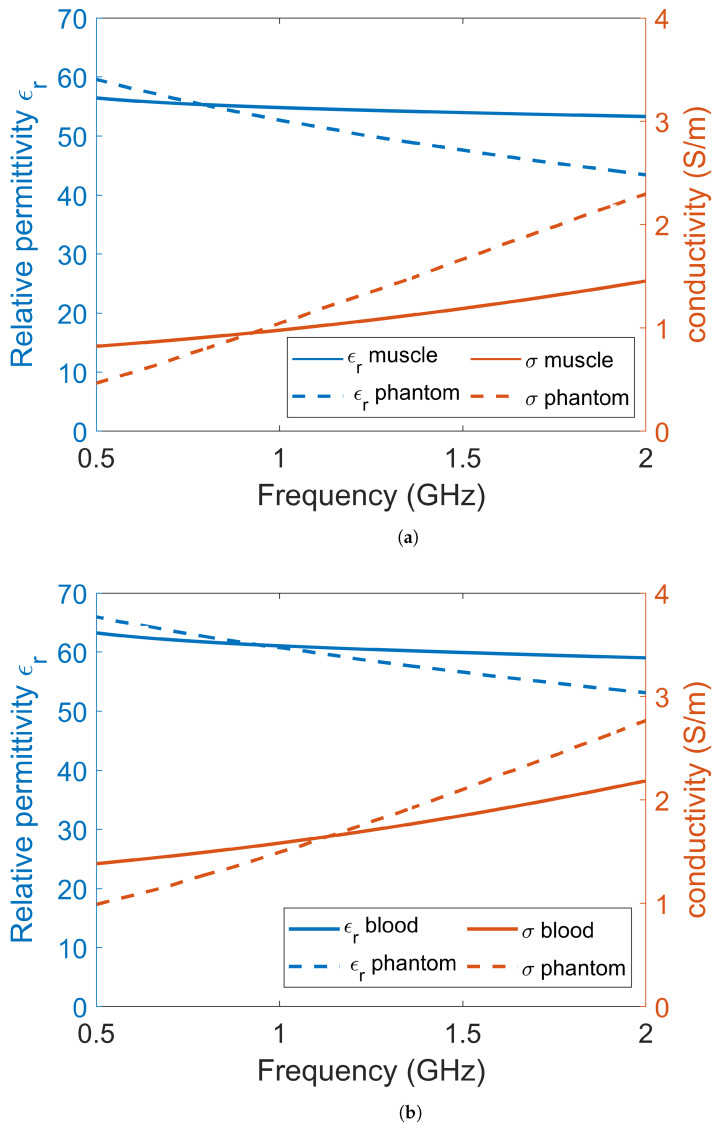
The dielectric data for (**a**) muscle tissue and (**b**) blood tissue. Each plot contains the permittivity and conductivity data from the literature, the measured dielectric values of the phantoms and the corresponding data that were used in the simulations.

The experiments and simulations were performed in a simplified environment consisting of only two tissues: muscle and blood. Muscle tissue was used to model the leg of a patient and blood was used to model the bleeding caused by a muscle rupture. Other tissues in the leg, such as bone, fat and skin, were omitted for simplicity. This simplified scenario should still be sufficient to show the proof of principle for the use of a lossy gel to mitigate the effects of multipath signals. Dielectric data for muscle tissue and blood tissue were obtained from [20] and are shown in Figure 1. For the experiments, muscle and blood phantoms were manufactured using water as a solvent, salt to control the conductivity, sugar to control the permittivity and agar to solidify the phantom [52]. The goal was to manufacture phantoms with properties that were as close as possible to published dielectric tissue data. However, with these substances, it was not possible to accurately mimic the dispersive behavior of real tissue over a wide frequency band. There are many other different substances with which to fabricate tissue-mimicking phantoms, such as oil in gelatin mixtures [53] and Triton X-100-based liquid mixtures [54], but these also come with the problem of inaccurately mimicking dispersive behavior compared to the tissue data. We decided to use water, salt and sugar because the manufacturing process is simple, the ingredients are harmless and the dielectric properties are good enough for a proof of concept. The permittivities and conductivities of the phantom and gel materials were measured using SPEAG’s Dielectric Assessment Kit (DAK). The dielectric properties used in the simulations were the same as the measured dielectric properties of the phantom material.

**Figure 2:**
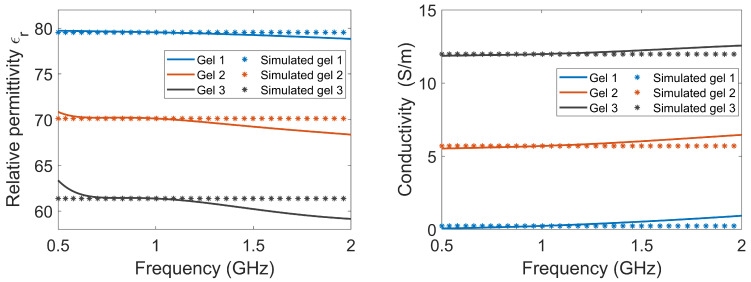
The relative permittivity and conductivity of the three lossy gels. The measured data of the gels, as well as the corresponding properties that were used in the simulations, are shown.

The antennae consisted of rectangular containers filled with lossy gel. Three different gels were manufactured to investigate the effects of the different attenuating properties of the gel: Gel #1 was made from regular tap water and 1.5 weight percent (wt%) agar; Gel #2 was made from a mixture of tap water, 1.5 wt% agar and 4 wt% NaCl (table salt); Gel #3 was made from a mixture of tap water, 1.5 wt% agar and 10 wt% NaCl. Figure 2 shows the measured dielectric properties of the gels, together with the corresponding properties that were used for the simulations. The measured properties of the gels are almost constant over the frequency band of interest. To simplify the numerical modeling, we used constant dielectric values that were equal to the measured properties at 1 GHz. This should not affect the validity of our conclusions.

### 2.2. Antennae, Antenna Array and Experimental Setup

**Figure 3:**
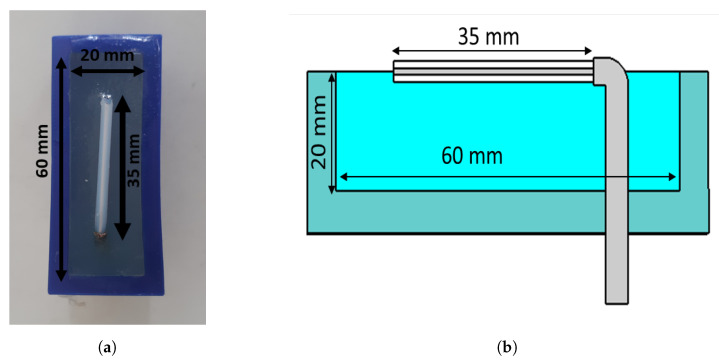
Monopole antenna with cup full of gel: (**a**) top view of antenna; (**b**) side view of antenna.

The antennae that we proposed for the muscle rupture detection application are shown in Figure 3a,b. The monopole antennae were manufactured by peeling off the outer conductor from a semirigid coaxial cable. The resulting radiating element had a length of 35 mm. The monopole was bent $90^{\circ }$, such that it could be mounted through a hole in the back of the plastic container, as seen in Figure 3b. The plastic containers had inner dimensions of 20 × 60 mm. The thickness of the plastic wall was 5 mm. The figure also shows the gel present inside the container. During the measurements, the antennae were applied in direct contact with the surface of the muscle phantom, such that the monopole elements were also in direct contact with the phantom. For the sake of maximizing the transmission into the leg, we did not want any lossy gel between the antenna and the skin, only behind and beside the skin to attenuate the outgoing multipath signals.

**Figure 4:**
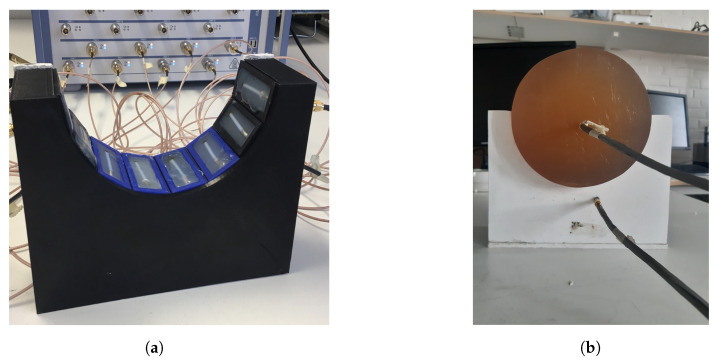
Measurement setup for probe measurements and image reconstruction: (**a**) semicircular antenna array consisting of 8 antennae; (**b**) muscle phantom in the antenna array, with probes.

Figure 4a shows the measurement system with the eight transmitting/receiving antennae. The antennae were mounted in a semicircular array with a 16-cm diameter and an angular spacing of $20^{\circ }$ between the individual antennae. The antennae were connected to a Rohde & Schwarz ZNBT8 16-channel vector network analyzer via flexible coaxial cables. This VNA has a dynamic range of up to 140 dB and operates within the frequency range of 9 kHz to 8.5 GHz, even though we only measured within the range of 0.5 GHz to 2.0 GHz. The measurements were taken using each antenna as a transmitter while the remaining antennae acted as receivers. In Figure 4b, the muscle phantom can be seen inside the antenna array. The phantom was slightly smaller than the array, with a diameter of 15.6 cm.

**Figure 5:**
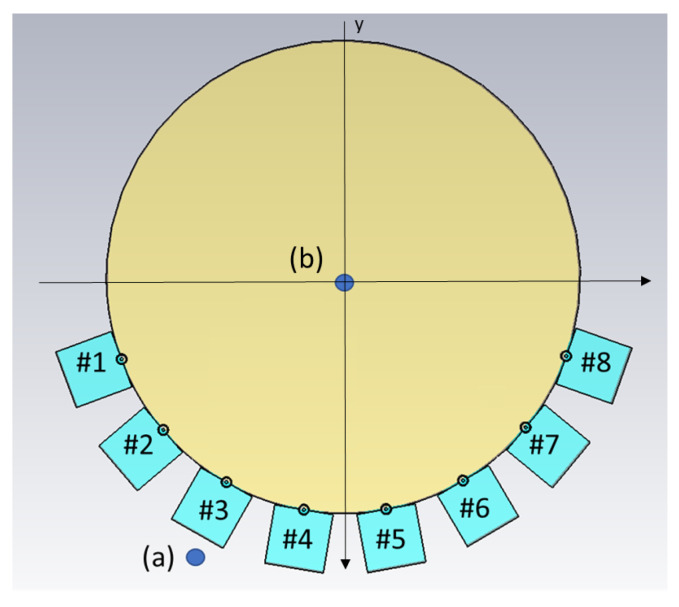
Schematic illustration of the simulation model, including the positions of the two field probes (**a**,**b**). Antennae are numbered #1–#8.

As already mentioned, the idea of this antenna design was to reduce multipath signals traveling outside the phantom, for example, surface waves. However, we did not want to sacrifice field strength inside the phantom, as this constituted the useful probing field. One set of experiments was conducted to investigate the amplitude of the fields, both outside and inside the muscle phantom, using different gels in the antennae. For this experiment set, two additional monopole antennae were used as field probes, both with lengths of 19 mm. The experiments were performed such that one antenna in the array transmitted, whereas the field probes were used to measure the amplitudes inside and outside the phantom, i.e., the transmission coefficients. The antenna and field probe arrangement that was used for both simulations and measurements is sketched in Figure 5. For both measurements and simulations, we chose to use the lowest antenna position within the array as the transmitter since this was where the phantom rested directly on the transmitting antenna and thus, had the best contact. Antenna #5 was used as the transmitting antenna and one of the two field probes was placed just behind Antenna #3, marked (a) in the figure. The other field probe was placed in the center of the muscle phantom, marked (b) in the figure. With these probes, we could determine how the field strengths inside and outside the phantom were affected by different gel properties. The measurements and simulations with Gel #1, Gel #2 and Gel #3 were conducted using this setup.

**Figure 6:**
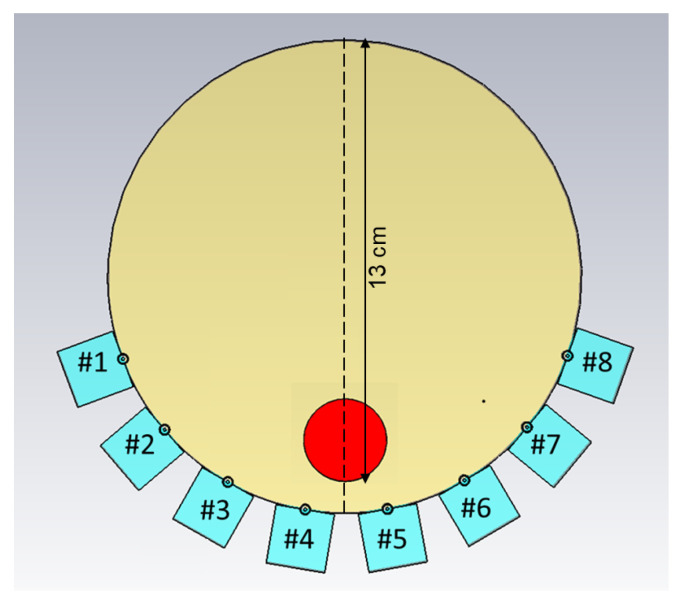
Schematic illustration of the measurement setup, including the position of the blood phantom, as well as the position of the transect cutting through the middle of the phantom. Antennae are numbered #1–#8.

**Figure 7:**
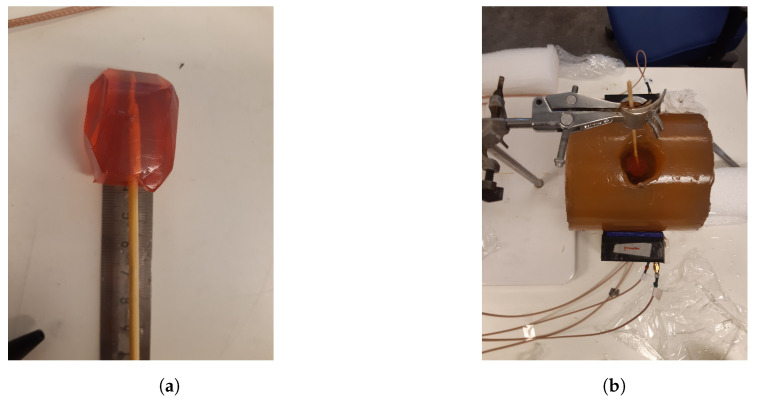
The blood phantom and muscle phantom that were used in the image reconstruction experiments: (**a**) blood phantom; (**b**) top view of the muscle phantom.

Another set of experiments was carried out for the purpose of image reconstruction. For this, a hole was carved into the muscle phantom to allow for the insertion of a blood phantom. The hole went 13 cm deep from the top of the phantom, as sketched in Figure 6. This hole was filled with a liquid muscle phantom of the same properties as the solid muscle phantom. This hole made it easy to make a priori measurements without the blood phantom being present. The blood phantom had a diameter of 4 cm and is shown in Figure 7a. Figure 7b shows the hole through which the blood phantom was inserted into the muscle phantom. A wooden stick was attached to the muscle phantom to facilitate the insertion and extraction of the blood phantom. The measurements were taken with and without the blood phantom present.

For the image reconstruction, the measurements were conducted such that the muscle phantom was placed in the antenna array and the measurements were repeated 15 times. Efforts were made not to touch or move anything within the setup between measurements. Then, the blood phantom was inserted into the hole in the muscle phantom until it touched the bottom and then 15 more measurements were taken. This procedure was repeated for both Gel #1 and Gel #3, amounting to a total of 60 measurements and 30 reconstructed images: 15 reconstructions with Gel #1 and 15 with Gel #3.

### 2.3. Simulations

Simulations corresponding to the field probe measurements were performed. The simulations were carried out in 3D and a cross-sectional image of the model is shown in Figure 5. In this figure, the muscle phantom is represented by a cylinder with a 16-cm diameter and the eight antennae, numbered #1–#8, are shown at the bottom of the image. The monopoles are shown as circles touching the muscle phantom and behind them are the quadratic-shaped gels. The plastic material of the containers was not included in this model. The dielectric properties of the simulated Gel #1, Gel #2 and Gel #3 are shown in Figure 2. The monopole antennae had the same dimensions as those in the measurements. The simulations were performed using the CST Studio Suite, Release Version 2020.07, in the frequency range of 0.5–3 GHz. In the same way as in the previous experiments, the simulations were performed using the two field probe antennae: one placed in the center of the phantom (a) and one placed just behind Antenna #3 (b).

### 2.4. Image Reconstruction Algorithm

The DMAS beamforming algorithm [30] was used to reconstruct the images. We used a multistatic configuration in which the signals between all possible antenna pairs were measured. To remove the early-time content, i.e., skin reflections, the measurements of the homogeneous muscle phantom were subtracted from the corresponding measurements with the blood phantom present.

## 3. Results

In this section, the results from the simulations and phantom experiments are presented. The results showed the effects of the conductivity in the antenna gels. Firstly, the simulation results are described in Section 3.1. We show the results of wave propagation inside and outside the muscle phantom using different lossy gels within the antennae. We also show examples of the simulations of S-parameter transmission data for Gel #1 and Gel #3. Secondly, the results from the experiments are shown and compared to the results from the simulations. Finally, the image reconstruction results are shown, based on the experimental data.

### 3.1. Wave Propagation Patterns Inside and Outside the Muscle Phantom

**Figure 8:**
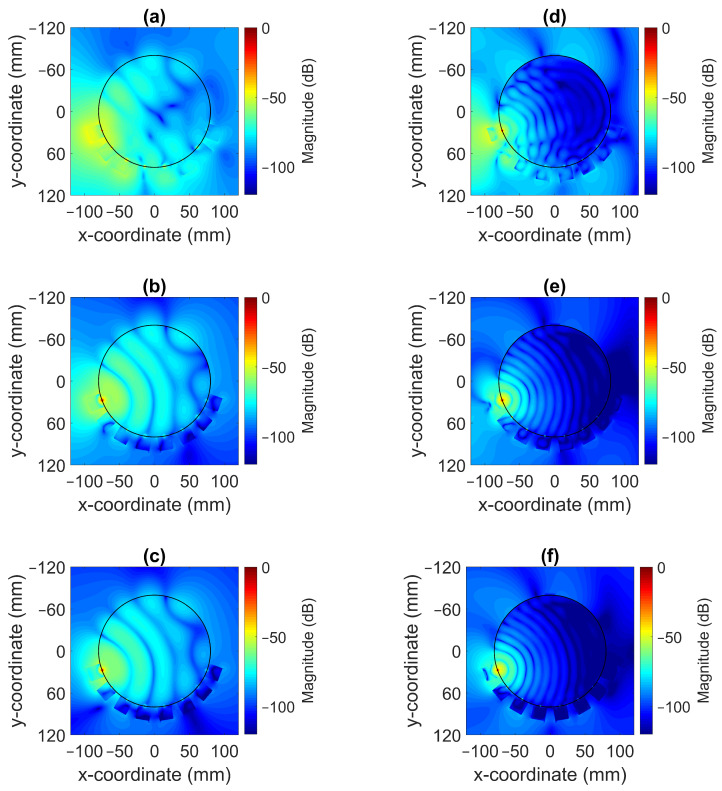
Simulated radiation patterns for a monopole antenna surrounded by coupling gels in contact with a muscle phantom for different conductivity values at 0.7 GHz ((**a**) Gel #1; (**b**) Gel #2; (**c**) Gel #3) and at 1.65 GHz ((**d**) Gel #1; (**e**) Gel #2; (**f**) Gel #3).

The magnitude of the electric field distribution was investigated using the simulation model shown in Figure 5 but without the field probes present. To elaborate, the cross-sectional images of the field patterns are shown for the frequencies of 0.7 GHz and 1.65 GHz in Figure 8. Antenna #1 was acting as transmitter. These images illustrate that it was possible to decrease the magnitude of the waves propagating outside the muscle phantom and around the antennae with a more lossy gel. For the lowest conductivity cases for Gel #1, as shown in Figure 8a,d, the wave patterns inside the phantom were rather chaotic and the field amplitudes outside the phantom were prominent. This effect was especially evident for the 0.7 GHz case. With increasing conductivity in the gel for Gel #2, as shown in Figure 8b,e, and the even higher conductivity in Gel #3, as shown in Figure 8c,f, it was seen that the field strengths outside the muscle phantom decreased and the wave fronts inside the phantom became more regularly shaped as semicircular wave fronts. We interpreted these results as meaning that the lossy gels helped to attenuate the multipath signals outside of the muscle phantom model. With weaker field strengths outside the muscle phantom, weaker fields were also coupled back into the phantom from directions other than the transmitting antenna itself and, therefore, less interference with the straight path propagation from the transmitting antenna occurred inside of the model. The effects of the multipath signal propagation were more apparent at 0.7 GHz than at 1.65 GHz due to the higher attenuation at higher frequencies.

**Figure 9:**
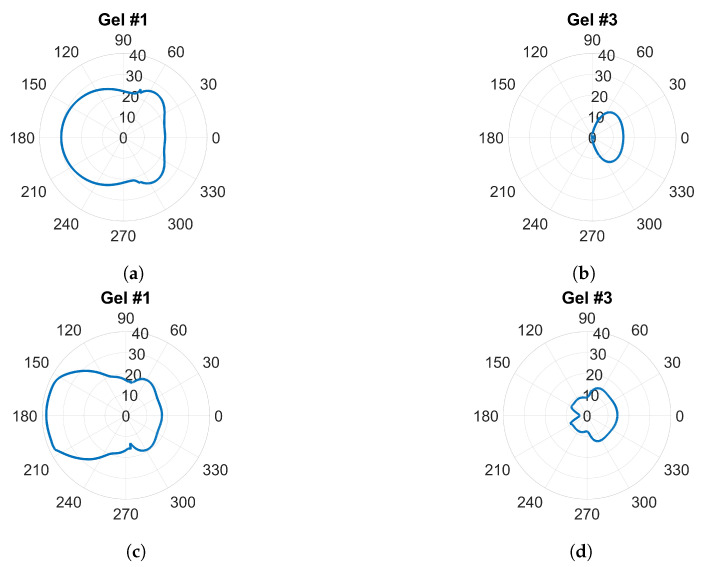
E-field sampled at a circle with radius λ around a single transmitting antenna: (**a**,**b**) at 0.7 GHz; (**c**,**d**) at 1.65 GHz.

Traditional radiation diagrams are not relevant to illustrate the directional properties of the antennae as they show far-field characteristics, so to further illustrate the damping effect of the gels, something similar to a radiation diagram was generated. The simulations were carried out as above at frequencies of 0.7 GHz and 1.65 GHz, but with only one transmitting antenna present and the other seven antennae removed. The magnitude of the electric field was sampled on a circle that was centered in the monopole element and had a radius of one wavelength (1λ) at the corresponding frequency. This field was then plotted in the radial diagram in Figure 9. The geometry was such that the plot corresponded to the H-plane of a radiation pattern and the angular coordinate was oriented so that $0^{\circ }$ in the plot was pointing in the same direction as the surface normal of the antenna at the antenna–phantom interface. For Gel#1 (Figure 9a,c), the field strengths behind the antennae were even higher than those in the muscle tissue in front of the antenna. Figure 9b,d show that for Gel #3, the E-field behind the antennae was significantly lower than that for Gel #1. In front of the antenna, the field was stronger; in fact, it was almost the same amplitude as for Gel # 1. This verified that the lossy Gel #3 effectively attenuated the waves traveling backward and sideways from the antenna.

### 3.2. Field Probe Simulations and Measurements

**Figure 10:**
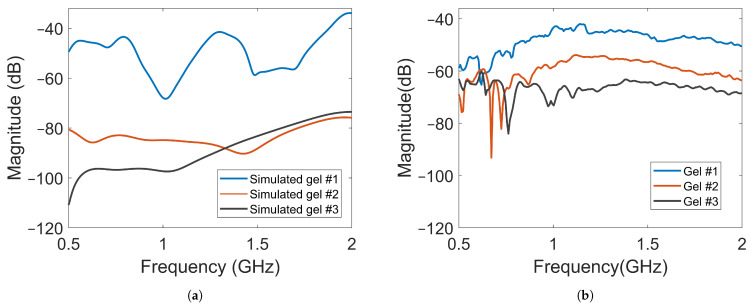
Magnitude of transmission coefficients between the transmitting antenna and Probe A for Gel #1, Gel #2 and Gel #3: (**a**) simulated transmission coefficients; (**b**) measured transmission coefficients.

The magnitude of the transmission coefficients that represented the electric field strengths at one point inside the muscle phantom and one point outside the phantom were measured and simulated. The positions are indicated in Figure 5. One antenna in the array was transmitting and the two field probes recorded the received data. Figure 10a shows the simulated magnitudes at probe location (A) as a function of frequency when Antenna #1 was acting as transmitter with the three different gels. Interestingly, the attenuation of the electric field (measured transmission coefficient) was not a linear function of the conductivity of the coupling gel. The decrease in signal level was much greater from Gel #1 to Gel #2 than from Gel #2 to Gel #3, indicating that it might approach a saturation point at which increased conductivity in the gels does not provide ever-increasing attenuation. Figure 10b shows the corresponding measured signals that were obtained from the field probe antenna positioned behind Antenna 3. For these measurements, the field strength around the muscle phantom decreased in magnitude as the conductivity of the material increased. Even though the levels were not exactly the same as in the simulations, the attenuation trend was similar, except at the frequencies in the range of 0.5 GHz to about 0.75 GHz, at which hardly any difference was seen between Gel #1 and Gel #2. However, for most of the frequency bands, the attenuation increased with increasing loss in the gel. Both the simulations and measurements agree that the more the conductivity in the gels increased, the more the leaking to the surrounding phantom decreased. Possible explanations for these differences include imperfections in the experimental setup and the computational model being overly simplified and not including any supporting structures, antenna cables, etc. The antenna gels had perfect contact with the curved surface of the muscle phantom in the simulations. In the experiments, there might be small air gaps between the edges of the flat antennae and the curved phantom surface. When the monopole element rose even slightly above the surface of the gel, this effect could be even more pronounced. The larger amplitudes in the field probe measurements than in the simulations suggested that this might be a plausible explanation.

Another complication was that the probe was placed in air and rather close to the transmitting antenna, where strong interference patterns could arise. This made the measurements more sensitive to misalignment between the simulations and experiments, for example.

**Figure 11:**
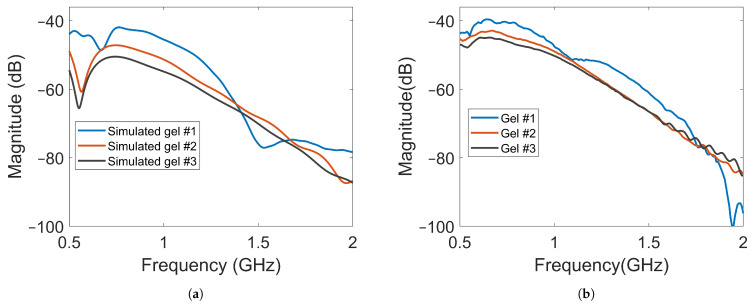
Magnitude of transmission coefficients between the transmitting antenna and Probe B for Gel #1, Gel #2 and Gel #3: (**a**) simulated transmission coefficients; (**b**) experimental transmission coefficients.

The amplitude of the electric field was also measured using a field probe in the center of the muscle phantom, as shown in Figure 5 with probe location (B). The purpose was to study whether the field strength inside the muscle phantom could also be attenuated by increasingly lossy gels. The waves inside the muscle phantom were the actual probing fields that were used to sense the scattering environment. Keeping them as high in amplitude as possible was therefore favorable from a measurement point of view. Figure 11a shows the simulated magnitudes of the electric fields at probe location (B). Here, Antenna #1 was also used as transmitter. A notable difference in the field strength outside the phantom was that the overall magnitude of attenuation between the different gels decreased to a much lesser degree, except in the region of 0.5–0.6 GHz. This meant that the increase in the conductivity of the gels could be implemented without greatly affecting the field strength inside the muscle phantom. Figure 11b shows the results of the measurements with the antenna in the middle of the muscle phantom. The measurement results agreed well with the simulated results for the field strength. These results indicate that this antenna design could be effective in attenuating multipath waves while preserving a high field amplitude inside the tissue.

#### S-Parameters

**Figure 12:**
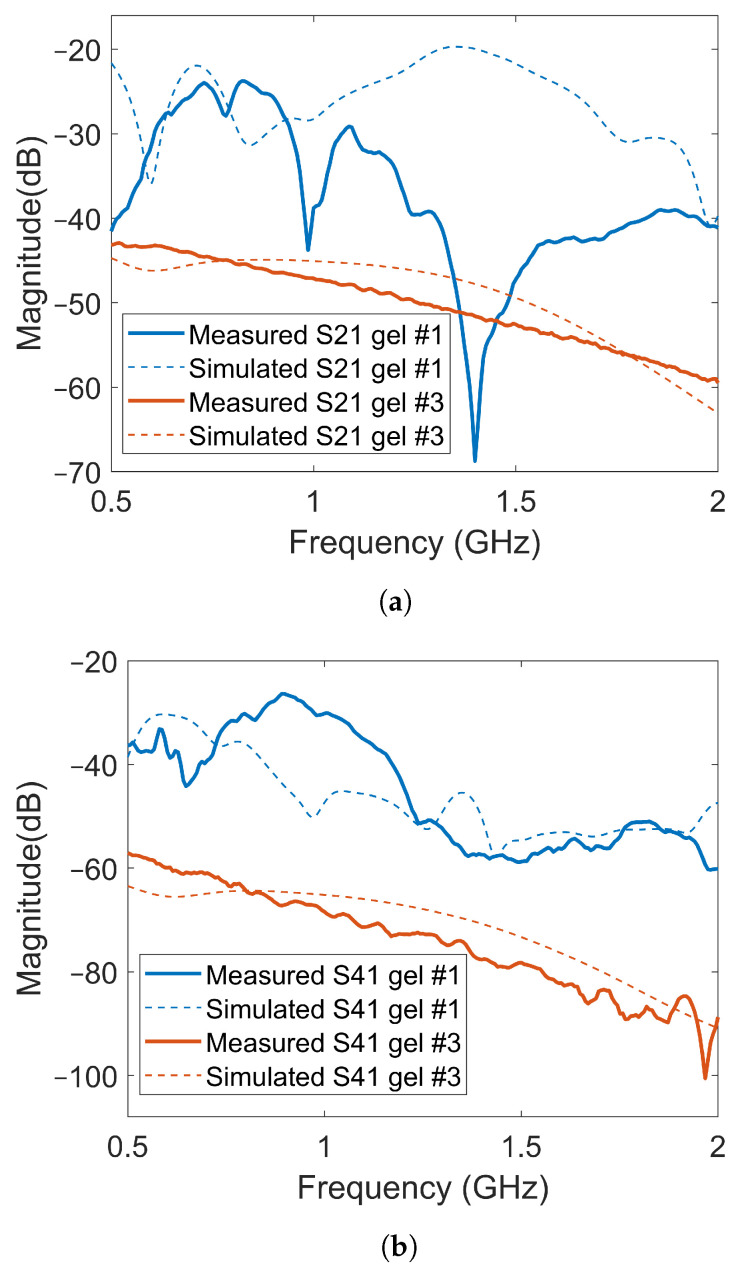
Comparison of transmission coefficients obtained from simulated and measured data for Gel #1 and Gel #3: (**a**) results for S21; (**b**) results for S41.

Figure 12 shows a few examples of the transmission coefficients (S21 and S41) for two of the previous cases with Gel #1 and Gel #3. Both measured and simulated data are shown. The first notable observation was that the overall amplitude for Gel #3 was lower than that for Gel #1 for both S21 and S41. Additionally, this figure shows the same behavior as that shown in Figure 10 and Figure 11, with the curve for Gel #3 being much smoother than that for Gel #1. Both the simulated and measured cases for Gel #1 showed a strongly alternating amplitude that appeared to be caused by the multipath waves interfering with the desired object response. As mentioned already, the amplitudes for Gel #3 were, in general, much lower in amplitude than those for Gel #1. As shown by the previous results, the field amplitudes inside the muscle phantom were preserved while the surrounding multipath signals were dampened. The lower amplitude was therefore a sign that the multipath signals had been removed and the scattering amplitudes from within the phantom had been preserved, which should be favorable in the subsequent signal processing and image reconstruction.

As shown in Figure 12, there were significant differences between the simulated and experimental data for Gel #1. Gel #1 had a low conductivity and, therefore, did not dampen the backward waves effectively. As a result, significant levels of multipath signals around the system that interfered with each other could be expected. The measured S-parameters, therefore, became quite unpredictable as even small changes in the experimental setup could lead to large fluctuations in the measured signals, making it difficult to obtain a good agreement between experimental and simulation data. To some extent, this also illustrated the importance of using a lossy gel, which reduced the levels of multipath signals and made the measurements more predictable. With Gel #3, the multipath signals were attenuated to a much larger degree, resulting in a better agreement between the measurement and simulation results.

### 3.3. Image Reconstruction Experiments

**Figure 13:**
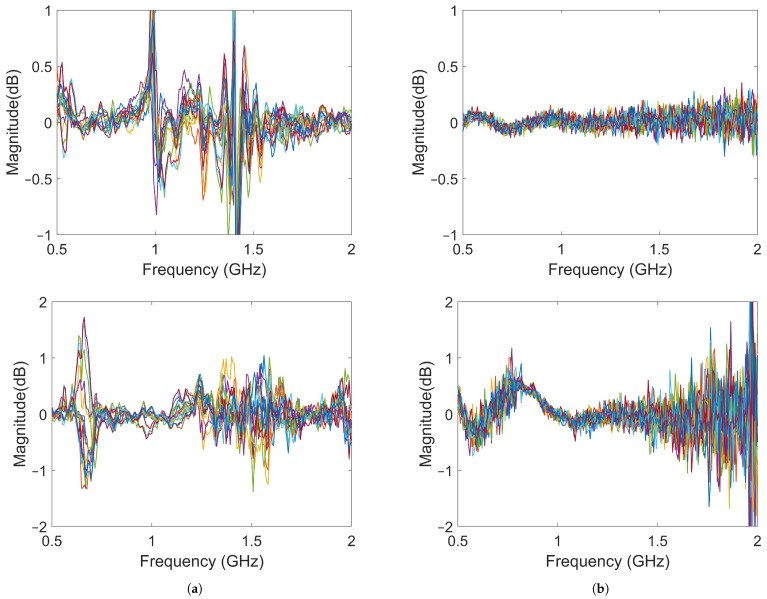
Differences between the object and baseline for 15 measurements: (**a**) results using Gel #1; (**b**) results using Gel #3. The upper images show the results for S21 and the lower images show the results for S41.

**Figure 14:**
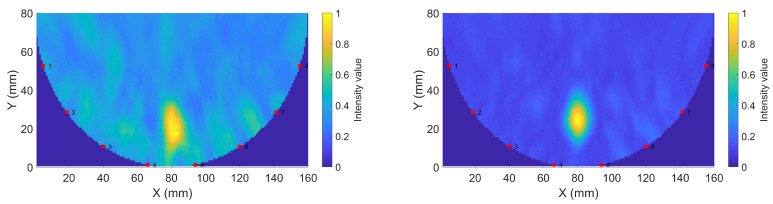

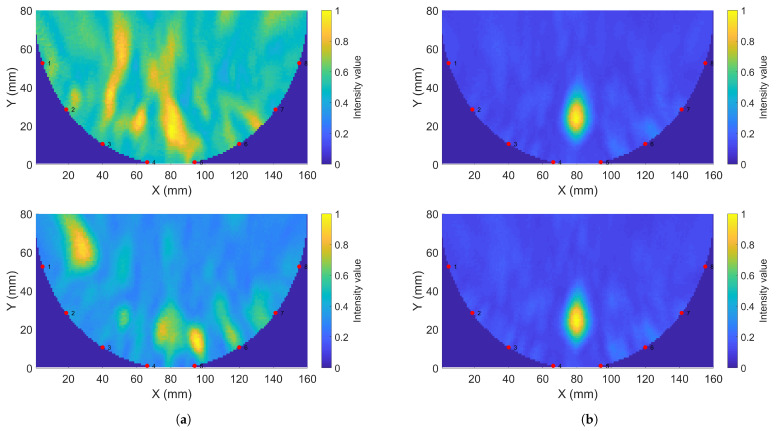
Sample reconstructions using phantom measurements: (**a**) Gel #1; (**b**) Gel #3.

**Figure 15:**
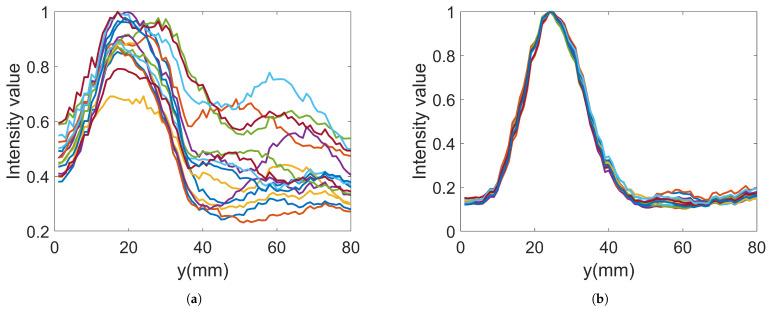
Reconstructed data along the line through the center of the imaging domain for 15 images: (**a**) Gel #1; (**b**) Gel #3.

In the imaging reconstruction experiments, two datasets were used, i.e., measurements with Gel #1 and those with Gel #3. Figure 10 and Figure 11 show that the results for Gel #2 were between those for Gel #1 and Gel #3 and in the spirit of keeping the rest of the paper as concise as possible, we chose to only use them in the imaging experiment. For each gel, 15 measurements were taken as a priori measurements and 15 measurements were taken with the blood phantom inserted into the muscle phantom. For each gel, 15 differential signals could then be calculated, i.e., the input signals for the DMAS algorithm. These are shown in Figure 13: Figure 13a shows the results for Gel #1 and Figure 13b shows the results for Gel #3. The two upper figures show the results for S21 and the two lower figures show the results for S41. As can be clearly seen, the results for Gel #3 were more stable but had an increasing level of noise with the increasing frequency. The results for Gel #1, however, showed a much more random variability, also for the lower frequencies. It was rather intuitive to conclude that a stable and repeatable input signal for the DMAS algorithm created a stable and repeatable image reconstruction. Figure 14 shows a few selected reconstructions that were obtained with the measured data for Gel #1 and Gel #3. The reconstructions from the measured data for Gel #3 showed fewer artifacts in the background, the three images were very similar, as can be seen by a visual inspection of the figure, and the blood phantom was reconstructed in the correct position. The reconstructions from the measured data for Gel #1, however, showed a much greater variability between the images. As can be seen in the figure, the upper image looked qualitatively quite good but with stronger artifacts close to the bottom edge compared to the images from the Gel #3 data. The other two images looked significantly different and some of the artifacts were even larger in amplitude than the reconstructed blood phantom target. To visualize all 15 image reconstructions, plots of the reconstructed amplitudes were drawn at the vertical line crossing through the center of the muscle phantom, see the dashed line in Figure 6. The reconstructed amplitudes are shown in Figure 15.

## 4. Discussion

In the work by Meaney et al. [36], it was demonstrated how multipath signals corrupt measured data within a microwave imaging system. By exploiting a lossy coupling bath, they successfully reduced the multipath signals and implemented a consistent imaging procedure. In the present work, the aim was to develop an imaging system for muscle rupture detection and diagnosis because of the need to immerse the entire leg in a big tank of lossy liquid is not practical and, in fact, prevents the widespread use of this technique as the system becomes too bulky and impractical to use. With our new design of a monopole antenna in front of a lossy gel to somewhat mimic the features of a lossy bath, the system becomes easier to handle. Antennae can be arranged in configurations that can be attached directly to the skin and allow the gels to dampen outgoing multipath signals that otherwise would cause artifacts in the measured data. In this work, both the simulated and measured data prove that the gels can successfully be used to reduce outgoing multipath signals. We noted some inconsistencies within the field probe data outside the antenna array, as shown in Figure 10. This was possibly due to imperfections in the experimental arrangement and an overly simplified simulation model. Further studies are needed to fully understand the sources of these discrepancies. However, the trend is clear and in agreement with the simulations that lossy gels do attenuate the outgoing signals.

A technique using directive antennae was suggested to maximize the energy that is coupled into the body while minimizing the energy that travels sideways and backward away from the body. In principle, this may also serve the same purpose of reducing multipath interference within the measurements. However, antenna designs are usually more complex and sophisticated compared to those for monopole antennae, which also suggests the need for a corresponding increase in the numerical modeling complexity. In the quest for fast quantitative imaging, a reconstruction method based on the discrete dipole method has been shown to exploit the simplicity of modeling a monopole antenna in a lossy bath [48]. A muscle rupture diagnostic method could very well be based on an efficient confocal imaging algorithm. However, as a long-term strategy, it seems reasonable to seek a solution that also works for fast quantitative imaging as the dielectric properties may contain important information with respect to diagnosing a patient. Therefore, a design that is based on monopoles is desired. The image reconstructions that were made using a lossy gel showed a significant improvement in the imaging quality over an almost lossless gel. We took great effort in performing the measurements on the muscle phantom without touching or moving anything within the experimental setup, yet the underlying microwave data, as well as the reconstructed images, showed a significant variability in the 15 reconstructed images. Performing measurements with the lossy gels, however, resulted in a very stable reconstruction of the 15 images, with the images looking almost identical. The only plausible explanation is that multipath signals were attenuated, which resulted in a significantly more stable imaging system.

## 5. Conclusions

We presented and investigated a novel design for antennae and an antenna array that is intended for the microwave imaging of the leg with the purpose of detecting and diagnosing muscle ruptures. The antennae consist of containers filled with a lossy gel and a monopole antenna mounted at the surface of the gel. During experimental measurements, the antennae were placed in direct contact with the muscle phantom and, consequently, the radiating elements were also in contact with the phantom. The reason for using the lossy gel on the back and side of the antennae was to attenuate the outgoing and sideways-moving waves and contribute to a reduction in multipath signals. The results showed that the field strengths outside the phantoms and antennae were significantly reduced when a lossy gel was used compared to when an almost lossless gel was used. The results also showed that the amplitudes of the transmission coefficients between the antennae decreased with increasing lossy in the gel; however, the field strength inside the muscle phantom only changed slightly. These results prove that the antennae are effective in reducing unwanted multipath signals while effectively preserving the amplitudes of the probing fields inside the object under investigation. The results obtained for the simulated and experimental data were in good agreement.

In repeated image reconstruction experiments using the DMAS, the lossy antennae resulted in significantly more stable and repeatable reconstructions than when an antenna with an almost lossless gel was used. We can conclude that a reduction in multipath signals made the signals less corrupted with unpredictable artifacts due to unwanted multipath scattering, particularly those from the region outside the muscle phantom, i.e., the antennae, cables, supporting structures, etc.

These results are promising, but the fine-tuning and optimization of the antenna characteristics may further improve the reduction in multipath signals and result in enhanced imaging capability. The implementation of a skin reflection removal algorithm that does not require a priori measurements of healthy tissue is also needed to make the technique more practically usable on patients. Future work should also include the use of more realistic phantoms or patients to investigate how image reconstruction is affected by other tissues, such as bone and fat, as well as how well blood phantoms of different sizes, shapes and locations can be reconstructed.

## Abbreviations

The following abbreviations are used in this manuscript:

2D	Two dimensional
3D	Three dimensional
DAS	Delay and Sum
DMAS	Delay, Multiply and Sum
